# 1027. Earlier Is Better: Progress Toward Decreased Time to Optimal Therapy and Improved Antibiotic Stewardship for Gram-positive Bloodstream Infections Through Use of GenMark Dx ePlex system

**DOI:** 10.1093/ofid/ofab466.1221

**Published:** 2021-12-04

**Authors:** Cameron White, Jeremy Meeder, Derek Moates, Hannah Pierce, Todd P McCarty, Rachael A Lee, Sixto M Leal

**Affiliations:** 1 University of Alabama at Birmingham, Birmingham, Alabama; 2 University of Alabama at Birmingham; Birmingham VA Medical Center, Birmingham, Alabama

## Abstract

**Background:**

The ePlex BCID Gram-Positive (GP) panel utilizes electrowetting technology to detect the most common causes of GP bacteremia (20 targets) and 4 antimicrobial resistance (AMR) genes in positive blood culture (BC) bottles. Rapid detection of intrinsic vancomycin resistance and acquired resistance genes (*mecA, mecC, vanA, vanB*) enables early optimization of antimicrobial therapy whereas early detection of common contaminants is expected to decrease unnecessary antibiotic utilization and hospitalizations.

**Methods:**

In this prospective study, aliquots of BC bottles with GP bacteria detected on Gram stain (GS) (n=101) received standard of care (SOC) culture and antimicrobial susceptibility testing (AST). Additionally, samples were evaluated with the BCID-GP panel but only SOC results were reported in the EMR and available to inform clinical decisions. Patients were excluded if the sample was a subsequent culture in a persistent episode of bacteremia (n=17) or if the assay failed (n=4). Chart review was performed to evaluate the expected impact of the BCID-GP panel on the time to organism identification, AST results, and optimization of antimicrobial therapy.

**Results:**

A total of 80 patients were included in the final analysis (Table 1). *S. epidermidis* was the most common bacteria identified, followed by *S. aureus*, and other coagulase-negative staphylococci. Thirty-nine patients with staphylococci (48.8%) had the *mecA* gene detected and 2 patients with *E. faecium* had the *vanA* gene detected. The BCID-GP panel saved a mean of 24.4 hours (h) to identification and 48.3h to susceptibility testing compared to standard methods across all patients. In 38 patients (47.5%), the BCID-GP panel result could have enabled an earlier change in antibiotic therapy. Table 2 highlights opportunities to optimize antimicrobial therapy 53.4h earlier for 16 (20%) patients with organisms expressing AMR genes, 29.2h earlier for 8 (10%) patients infected with organisms, such as streptococci, with very low resistance rates, and to stop antimicrobial therapy 42.9h earlier for 14 (17.5%) patients with contaminated blood cultures.

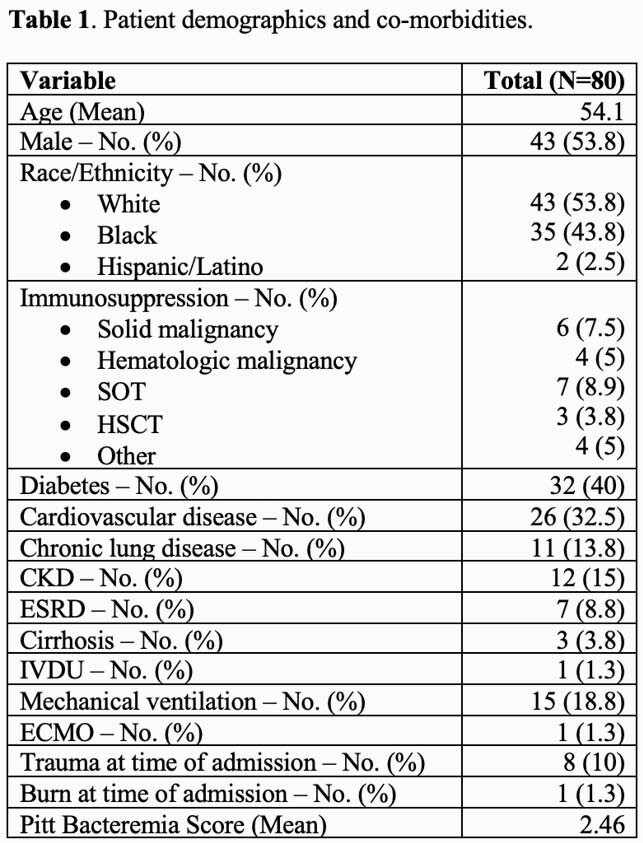

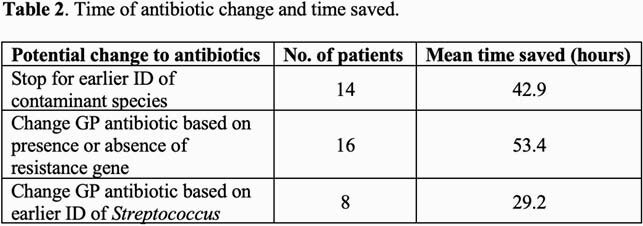

**Conclusion:**

The BCID-GP panel could have enabled earlier optimization or stopping of antibiotics in many patients with significant time savings compared to standard laboratory methods.

**Disclosures:**

**Todd P. McCarty, MD**, **Cidara** (Grant/Research Support)**GenMark** (Grant/Research Support, Other Financial or Material Support, Honoraria for Research Presentation)**T2 Biosystems** (Consultant) **Sixto M. Leal, Jr., MD, PhD**, **Abnova** (Grant/Research Support)**AltImmune** (Grant/Research Support)**Amplyx Pharmaceuticals** (Grant/Research Support)**Astellas Pharmaceuticals** (Grant/Research Support)**CNINE Dx** (Grant/Research Support)**GenMark Diagnostics** (Grant/Research Support, Other Financial or Material Support, Honoraria- Research Presentation)**IHMA** (Grant/Research Support)**IMMY Dx** (Grant/Research Support)**JMI/Sentry** (Grant/Research Support)**mFluiDx Dx** (Grant/Research Support)**SpeeDx Dx** (Grant/Research Support)**Tetraphase Pharmaceuticals** (Grant/Research Support)

